# Reduced Salivary Mucin Binding and Glycosylation in Older Adults Influences Taste in an In Vitro Cell Model

**DOI:** 10.3390/nu11102280

**Published:** 2019-09-24

**Authors:** Rose-Anna G. Pushpass, Nicola Pellicciotta, Charles Kelly, Gordon Proctor, Guy H. Carpenter

**Affiliations:** 1 Salivary Research-Centre for Host-Microbiome Interactions, Faculty of Dentistry, Oral and Craniofacial Sciences, King’s College London, London SE1 9RT, UK; 2 Cavendish Laboratory, University of Cambridge, Cambridge CB3 0HE, UK

**Keywords:** saliva, taste, ageing, rheology, mucin

## Abstract

Background: Taste loss is a significant problem in older adults, affecting quality of life and nutrition. Altered salivary rheology and loss of mucin function may contribute to taste loss by reducing mucosal defences in the oral cavity, impairing sensitivity to oral stimulants. This study aimed to investigate the effects of salivary rheology on taste loss in ageing. Salivary mucin glycosylation and binding to the oral epithelium was investigated in older and younger adults. A cell-based model was utilised to consider the role of saliva in taste loss. Methods: Human subjects aged >60 years (*n* = 25) and 18–30 (*n* = 30) provided saliva samples which were analysed for viscosity, mucin composition and mucin binding to oral epithelial cells (TR146/MUC1). Oral epithelial cells (TR146/MUC1 and SCC090) provided models for taste receptor activation. Results: Reduced levels and sialylation of MUC7 were evident in saliva of older adults which may lead to reduced viscoelasticity, while viscosity is unaffected. Impaired muco-adhesion of saliva from older adults was also observed. Saliva from older adults facilitated the bitter taste receptor activation less well than saliva from younger adults. The causes of taste dysfunction in older adults are unknown, but this study supports a role of saliva in facilitating the activation of taste receptors.

## 1. Introduction

There is a link between age and the decline of taste acuity, especially for bitter taste compounds [1,2]. In ageing, loss of taste can result in malnutrition and diet-related disorders that are highly prevalent in adults aged >60 years old and represent a significant public health risk [3,4].

Saliva is present in the oral cavity and constantly bathes the taste buds on the tongue where it can interact with sensory stimulants and play a role in taste, smell, and chemo-sensation [5,6]. Ageing results in changes in the physical properties of saliva, including reduced viscoelasticity [7]. This is likely because of the alteration in mucin composition, with reduced total levels of MUC7 [7]. Muco-adhesion of saliva could also play a role in the retention of tastants on the tongue which may affect taste receptor activation [8]. Certain muco-adhesive polysaccharides can prolong the perception of taste molecules likely by prolonging tastant retention and access to taste receptors [9,10].

Changes in the saliva of older adults could therefore impact upon taste function including the ability to taste bitter compounds. Bitter tastants are generally hydrophobic or lyophilic in nature and therefore their ability to activate taste receptors may depend on interactions with mucins in saliva. Furthermore, increased hydrophobicity of a compound leads to a reduced ability to form hydrogen bonds with aqueous saliva and thus the ability to reach taste receptors may be enhanced as the hydrophobic groups repel the water and are driven into the non-polar environment of the receptor membrane [11,12].

Previous studies showing tastant-receptor interactions using in vitro heterologous expression models have often used extra-oral cell lines and failed to consider the effect of saliva [13,14,15]. Saliva plays an important role in taste function since xerostomia and dry mouth is frequently coupled with reduced taste sensitivity [16,17,18]. In addition, morphological changes in taste buds, coupled with reduced taste sensation, has been observed in salivary gland excised rats [19,20]. The taste buds are constantly bathed in whole saliva, or in the case of the posterior tongue, in secretions from the von Ebner salivary glands [21,22]. Saliva solubilises tastants and transports them to the taste buds where they can activate taste receptors [23,24]. Tastants in solution can only access taste receptors via diffusion through the salivary mucosal pellicle on the tongue [5,25]. For this reason, the model created in this study utilised cell lines originating from the human oral cavity and studied the effects of saliva on taste receptor activation. Both cell lines were shown to facilitate salivary MUC5b binding, similarly to the oral epithelium in vivo. Transfection of TR146 MUC1 cells, with TAS2R38 and Gα16Gust44 cDNA provided a model for the study of tastant-receptor interactions. TAS2R38 is a bitter receptor expressed by type ii taste cells and responsible for sensation of bitter compounds containing a thiouracil ring such as Phenylthiocarbamide (PTC) and 6-n-propylthiouracil (PROP) [26,27]. In addition, SCC090 cells provided an alternative model of the taste receptor function. This cell line is derived from the human tongue epithelium and, in this study, was shown to constitutively express several TAS2R receptors, required for the bitter taste function.

## 2. Subjects and Materials

### 2.1. Study Group

Thirty younger (26 female, 4 males, mean age 24.3 +/− 0.4 years old) and 24 older (18 female, 6 males, mean age 72.4 +/− 1.8 years old) participants took part in this study and were detailed in a previous study [7]. Smokers, pregnant or breast-feeding women and those with swallowing difficulties were excluded. An oral health check was conducted by a trained dentist at least 5 min prior to beginning saliva collections to ensure that all volunteers were free of oral disease. Participants with acute disease and infectious illnesses were excluded, however, volunteers were not excluded based on medication use. This was to ensure that the study group would be representative of older adults in the general population since the majority of older adults do take one or more medications. Older subjects (*n* = 11) took more (1.9 +/− 0.7 vs. 0.3 +/− 0.2) prescribed medicines than the younger group but similar amounts of over the counter medicines (1.2 +/− 0.6 vs. 1.3 +/− 0.6) in the younger group (*n* = 8). Medications included anti-depressants, anti-hypertensives, anti-hyperlipidemic, anti-anxiety, painkillers, and antacids.

This study was carried out in accordance with the recommendations of King’s College London Guidelines on Good Practice in Academic Research. The protocol was approved by the King’s College London Biomedical Sciences, Dentistry, Medicine and Natural and Mathematical Sciences research ethics committee (BDM RESC), application reference: BDM/12/13-130. All subjects gave written informed consent in accordance with the Helsinki Declaration. 

All volunteers had unstimulated salivary flow rates above 0.1 mL/min, considered in the healthy range (data published previously—[7]), however, some participants had salivary flow rates which gave lower saliva sample volumes that were insufficient for all of the analytical experiments. As such, for some analyses, a smaller sub-set of samples from the same study group were used.

### 2.2. Saliva Collection

Volunteers were asked to donate unstimulated whole mouth saliva (UWS) samples. Collections were carried out in the Mucosal Biology department of King’s College London in the morning (between 8 am and 12 pm) and used the passive drooling technique, for 2 min. Subjects were asked to refrain from eating or drinking at least 2 h before samples were taken. Volunteers were sitting upright and asked to lean forward and allow saliva to collect into a sterile falcon tube. Samples were stored on ice immediately after collection, aliquoted to minimize freeze thaw cycles, and stored at −80 °C. 

### 2.3. Analysis of Salivary Viscosity Using Differential Dynamic Microscopy (DDM)

UWS samples were thawed on ice and mixed with 1 um fluorescent beads (2%) coated with polyethylene glycol (PEG) [28] and sealed under a glass cover slip on a microscope slide using UV-cured glue. Slides were imaged in a bright field on a Ti-E inverted microscope (Nikon, Tokyo, Japan) using a 40X magnification equipped with a 40 × dry objective (NA = 0.95; Nikon). High speed videos were then recorded using a CMOS camera (model No. GS3-U3-23S6M-C; Point Grey Research/FLIR Integrated Imaging Solutions (Machine Vision), Richmond, BC, Canada), recording a 10 s video with a resolution of 1200 × 1200 pixel (px) at 160 fps. Images were taken from three distinct regions of each sample and an average taken to control for heterogeneity of the WS samples. Images were acquired at room temperature (average 25 °C). Diffusion coefficient (D) of the suspended fluorescent beads of radius (r) was measured using Differential Dynamic Microscopy (DDM) (31). From the diffusion coefficient (D) it was possible to extract the viscosity of the sample using the Stokes–Einstein equation: Viscosity (Pa·s) = Kb T/(6 π D r), where Kb is the Boltzmann constant, T is the temperature, and r is the radius of the particles. In DDM, image sequences are analysed via a combination of image differences and spatial Fourier transforms to access information equivalent to that obtained by means of light scattering techniques as Dynamic Light Scattering (DLS) [29]. The DDM approach is particularly useful when samples of interest are available only in very small volumes, as in this study, or as thin quasi two-dimensional materials (e.g., cell cultures [30]) that are not compatible with traditional dynamic light scattering approaches. 

### 2.4. Total Protein Concentration

The bicinchoninic acid assay (BCA assay) kit (BioVision, Milpitas, CA, USA) was used to determine the total protein concentration of samples. Bovine Serum Albumin was used as a standard, 2–0.025 mg/mL, with water blank. Un-diluted saliva samples were added to a 96-well plate in duplicate, followed by the addition of BCA reagent. The absorbance at 540 nm was measured using the iMark Microplate Absorbance Reader (BIORAD, UK). Protein concentrations of samples were calculated with a standard curve created by plotting on a linear graph the standard concentration (mg/mL) against absorbance (nm). A linear equation was generated from the graph and used to calculate sample protein amounts from absorbance readings.

### 2.5. Gel Electrophoresis

Sodium Dodecyl Sulphate Polyacrylamide Gel Electrophoresis (SDS-PAGE) was used to analyse protein composition of samples. Samples were prepared in NuPAGE lithium dodecyl sulphate sample buffer (LDS) sample buffer (25%) (Life Technologies, Paisley, UK) under reducing conditions (50 mM dithiothreitol (DTT)). Samples were boiled for 3 min at 100 °C and 50 µg total protein loaded onto the gel (Xcell electrophoresis unit (Life Technologies)). Electrophoresis with MES SDS running buffer (50 mM MES, 50 mM Tris Base, 0.1% SDS, 1 mM EDTA, pH 7.3 (Life Technologies)) was at constant voltage (200 v) for 32 min. A control sample of un-stimulated whole mouth saliva (UWS) from one healthy donor (aged 27 years) was included in each gel to allow for normalizing between gels.

### 2.6. Western Blotting 

Following SDS-PAGE, resolved proteins were transferred electrophoretically to nitrocellulose membranes in an Xcell vertical electrophoresis unit (Invitrogen, Carlsbad, USA) with transfer buffer (NuPage Transfer Buffer (Invitrogen, Carlsbad, CA, USA), 10% Methanol, UHQ water). Transfer was at 30 volts constant and 200 amps for 60 min. 

Following transfer, membranes were blocked for 1 h in 2%–5% (w/v) non-fat milk powder (NFM) (Marvell, Premier Foods, London, UK) dissolved in TBS-T solution (Tris-Buffered Saline, 20 mM Tris, 0.9% NaCl and 1% Tween 20, pH 7.6). After washing membranes in TBS-T, they were incubated in primary antibody solution at 1:500 dilution in TBS-T (EU-MUC5Bb or EU-MUC7 Novus Biologicals, Abingdon, UK) for 1 h at room temperature or overnight at 4 °C. Blots were washed in TBS-T and then incubated in secondary antibody at 1:10,000 dilution in TBS-T (Goat-anti-mouse igG, Sigma, Dorset, UK) for 1 h at room temperature. 

Bound antibody was visualised using chemiluminescent substrate Luminata Crescendo Western HRP (Merck Millipore, Watford, UK) and blots were exposed to X-ray film (Photon Imaging Systems Ltd, Swindon, UK) for 30 s to 5 min. The JPI automatic X-ray film processor model JP-33 (Jungwon Precision Industries Ltd. Seoul, Korea) was used to develop films with RG rapid X-ray developing solution and X-ray fixer (Champion Photochemistry, Kuala Lumpur, Malaysia). Films were processed for 90 s at 37 °C followed by fixative for 3 min and drying at 50 °C. All exposure and developing steps were conducted in dark conditions. 

### 2.7. Detection of Sialic Acid

To detect total sialic acid residues in saliva, UWS samples were electrophoresed by SDS-PAGE and transferred to nitrocellulose as described above. After transfer, membranes were washed in TBS-T (Tris-Buffered Saline, 20mM Tris, 0.9% NaCl and 1% Tween 20, pH 7.6) for 30 min at room temperature. Membranes were probed with biotinylated Sambucus nigra agglutinin (SNA, 0.05 μg/mL) for detection of α-2, 6 linked sialic acid, and biotinylated Maackia amurensis leukoagglutinin II (MAL II, 0.4 μg/mL) was for detection of α-2, 3 linked sialic acid (Vector Laboratories, Burlingame, CA, USA). Both lectins were diluted in TBS-T and incubation was at room temperature for 60 min. Following washing in TBS-T, 6 × 2.5 min, signal from bound lectins was detected using horseradish peroxidase linked streptavidin (Vector Laboratories). Lectin signal was visualised using chemiluminescent substrate as described above. 

### 2.8. Semi-Quantification of Immuno-Blots

Densitometry was conducted to semi-quantify band intensities of Western blots. Western blot films were scanned, and images were imported into Image J version 1.46 (NIH, Bethesda, MD, USA) for semi-quantification of the band pixel intensity.

### 2.9. Cell Culture

Experiments were carried out using the TR146 buccal epithelial carcinoma cell line stably transfected to express human MUC1 (a kind gift from Professor Martine Morzel, INRA, Centre des Sciences du Goȗt et de l’Alimentation, Dijon, France) and SCC090 squamous cell carcinoma cell line derived from the base of the tongue. TR146/MUC1 and TR146 cells were cultured in DMEM/F12 medium (Gibco), supplemented with 15% foetal bovine serum (FBS) and 100 units/mL penicillin, 100 μg/mL streptomycin in T175 flasks (Life Technologies, Carlsbad, CA, USA). SCC090 cells were cultured in Eagle’s Minimum Essential Medium (EMEM) supplemented with non-essential amino acids (L-Alanine 0.89 g/L, L-Asparagine 1.5 g/L, L-Glutamic Acid 1.47 g/L, Glycine 0.75 g/L, L-Proline 1.15 g/L, L-Serine 1.05g/L), 10% FBS and 1% gentamycin (all Sigma, Dorset, UK). TR146 cells were sub-passaged every 3 to 4 days with medium changes every 2 days. SCC090 cells were sub-passaged every 5 days with medium changes every 2 days. Cells were incubated at 37 °C, 5% CO_2_. 

### 2.10. Mucin Muco-Adhesion Assay

For the assay, TR146 cells were seeded at a density of 5 × 10^5^ cells/mL and SCC090 were seeded at a density of 1 × 10^6^/mL, both in 12-well culture plates, incubated for 24 h until >80% confluency was achieved. A layer of saliva was added onto cells by incubating sub-cultures for 2 h with saliva diluted 1:1 into growth medium, as previously described [31]. Saliva samples were clarified by centrifugation at 2000× *g* for 10 min at 4 °C before depositing onto the cells. Following the incubation period, samples were washed twice with PBS to clear any non-adsorbed salivary mucin. Cells were lysed using a modified RIPA buffer (50 mM Tris-HCl pH 7.4, 150 mM NaCl, 1 mM EDTA, 1% Triton X-100, 1% sodium deoxycholate, 0.1% SDS) containing protease inhibitor cocktail (Sigma-Aldrich, Dorset, UK). The lysates were collected and centrifuged at 13,300× *g* for 10 min at 4 °C to eliminate cell debris. The supernatant was used for Western blotting of bound mucins (performed as described above). Lysates were stored at −20 °C between analyses and kept on ice during experimental work to prevent degradation by proteases. 

### 2.11. Transfection and mRNA Analysis 

#### 2.11.1. Purification of Plasmid DNA

hTAS2R38 PAV plasmid DNA was a kind gift from Professor Maik Behrens, Leibniz-Institute for Food Systems Biology at the Technical University of Munich. The Gα16Gust44 chimeric construct was a gift from Professor Takashi Ueda, Division of Cellular Dynamics, National Institute for Basic Biology (NIBB), Okazaki, Japan. Preparation of this construct was described previously [32].

XL1-blue competent *E. coli* (Agilent, Santa Clara, CA, USA) was transformed according to the manufacturer’s instructions and plasmid DNA was purified using the QIAprep Spin Miniprep Kit (QIAGEN, West Sussex, UK) following the manufacturer’s instructions. Mini-prep DNA samples were digested with HINDIII and NOTI restriction enzymes (Promega, Southampton, UK), used according to the manufacturer’s instructions. Briefly, 10–20 U of restriction enzyme was added to 1 μg of plasmid DNA with the appropriate buffer. Mixtures were incubated at 37 °C for 60 min. DNA fragments of the expected size were confirmed by agarose gel electrophoresis. 

Subsequently, DNA was purified for use in transfections using GeneJET Endo-Free Plasmid Maxiprep Kit (Thermo Fisher Scientific (Life Technologies), Paisley, UK). 

#### 2.11.2. Primer Design

To minimise the possible amplification of genomic DNA, primer pairs for the determination of the transfection of the human cell lines were designed, where possible, so that at least one primer in a pair would span an intron-exon boundary (Table 1). To design primer sets, the Roche universal probe library assay design centre software was used (https://lifescience.roche.com/). Primer sets were assessed for specificity to the target gene using the NCBI nucleotide BLAST software (http://blast.ncbi.nlm.nih.gov/Blast.cgi). 

### 2.12. DNA Agarose Gel Electrophoresis 

DNA was analysed using agarose gel electrophoresis. Electrophoresis grade agarose (0.8–2% w/v) (Sigma–Aldrich, Dorset, UK) was dissolved in TAE buffer (0.004 M Tris-acetate, pH 8.0; 1 mM EDTA) with heating. The gel was cooled to 55 °C, 10 μL of GelRed (Biotium, Hayward, CA, USA) was added and the mixture was poured into a cast. DNA samples were prepared by addition of loading buffer comprised of 0.04% (w/v) bromophenol blue, 0.04% (w/v) xylene cyanol and 5% glycerol. Electrophoresis was at 120 V for 1 h. Bromophenol blue and xylene cyanol were used as tracking dyes. DNA molecular weight markers (1 kb DNA ladder, 100 bp DNA ladder; New England Biolabs, Ipswich, MA, USA) were run alongside the DNA samples to allow for determination of approximate DNA fragment size. Gels were imaged under UV light and images were taken by using a gel documentation system (Syngene).

### 2.13. Determination of DNA/RNA Concentration

DNA/RNA concentrations were determined using the Nanodrop 2000 (Labtech International Ltd., Heathfield, UK). DNA samples with OD 260/OD 280 ratio between 1.8–2.0 were used in experimental work. 

### 2.14. Transfection of TR146 and TR146/MUC1 Cells

For the expression of TAS2R38/Gα16Gust44, TR146 and TR146/MUC1 cells were transfected using plasmid DNA prepared as described above. 0.3 μL of LipoJet transfection reagent (Promega, Southampton, UK) was added to the buffer supplied (5 μL per well) and the mixture was vortexed for 5 s to mix. Endotoxin-free TAS2R38 and Gα16Gust44 DNA (100ng:50ng per well) was added to the mixture, vortexed for 5 s and incubated for 10 min at room temperature. Next, 1 × 10^6^/mL cells were re-suspended in complete DMEM/F12 Ham media and 50 µl/well seeded in a 96-well plate before the transfection reaction mixture was added immediately. Cells were incubated for 24 h at 37 °C, CO_2_ (5%) before use in calcium assays. A mock transfection, using an empty vector, was conducted alongside transfection using DNA of interest as a negative control. Empty pcDNA 3.1 + vector (Invitrogen, Carlsbad, CA, USA) was purified as described above and transfected into cells using identical conditions to the TAS2R38/Gα16Gust44. 

### 2.15. RNA Extraction

RNA was extracted from cells using the GeneElute Mammalian Total RNA Kit (Sigma, Dorset, UK) following the manufacturer’s instructions. Cells were seeded 24 h prior to RNA extraction at 2.5 × 10⁵/mL in 6 well tissue culture plates. For transfected cells, RNA extraction was performed 24–48 h after transfection. DNase digestion was performed to eliminate gDNA from the RNA preparations. RNA was eluted with 50 μL tris solution (10 mM, pH 8.5). The concentration was determined using the NanoDrop and RNA was used for RT-PCR experiments. 

### 2.16. Reverse Transcription

cDNA used in PCR was synthesised from RNA preparations using reverse transcription. In a PCR reaction tube, a RT reaction mixture (10 μL total) was created, comprised of 1 μL of oligoDTs (50 μM, Sigma, Dorset, UK), 1 μL of DNTPs (aqueous solution of dATP, dCTP, dGTP and dTTP, each at a final concentration of 10 mM, Thermo Scientific, Paisley, UK), 1 µg RNA, and molecular biology grade water (Thermo Scientific, Paisley, UK). The mixture was incubated for 5 min at 65 °C before rapid chilling on ice for 1 min. Next, 1 μL superscript iv reverse transcriptase (Thermo Scientific, Paisley, UK) was added, with 4 μL buffer (supplied with the superscript kit), 1 μL DTT and 4 μL molecular biology grade water. The mixture was then incubated at 25 °C for 5 min, then at 50 °C for 60 min, and finally the reaction was inactivated by incubating at 70 °C for 15 min. The concentration of cDNA samples was determined using the NanoDrop and samples were stored at –80 °C between experiments. 

### 2.17. PCR Procedure 

#### 2.17.1. Polymerase Chain Reaction (PCR) 

Polymerase Chain Reaction (PCR) was conducted by mixing 10 ng of plasmid DNA, in 50 μL total of PCR reaction mixture comprised of dNTPs (deoxyribonucleotides: dATP, dCTP, dGTP and dTTP each at 0.2 mM); 2.5 mM MgCl_2_ (Promega, Southampton, UK); 10X reaction buffer [200 mM Tris-HCl (pH 8.8 at 25 °C), 100 mM (NH4)2SO4, 100 mM KCl, 1% (v/v) Triton X-100, 1 mg/mL BSA]; 2.5U PfuUltra™ HF DNA polymerase (Stratagene, La Jolla, CA, USA); 10 pmol of 5′ phosphorylated sense and antisense oligonucleotide (Sigma–Aldrich, Dorset, UK). The template was denatured for 30 s at 95 °C. Thirty-five denaturation-annealing-extension cycles were conducted. After the completion of the cycling, a final incubation was conducted for 5 min at 72 °C for final annealing and extension.

#### 2.17.2. FLEX Station Intracellular Calcium Measurements

Intracellular calcium measurements were conducted using fura-2/fluo-4 staining of cells. For the assays, TR146 MUC1 cells were seeded on black, clear bottom 96-well culture plates (Greiner Bio-One Ltd, Gloucester, UK), at a density of 5 × 10^5^ cells/mL. SCC090 were seeded at a density of 1 × 10^6^/mL, incubated for 24 h until >80% confluency was achieved.

TR146/MUC1 and SCC090 cells were pre-incubated with 50 μL/well of a fura-2-AM solution consisting of 5 μL fura-2-AM/fluo-4AM (Life Technologies, Paisley, UK) (prepared at a concentration of 2.5 mM in 50% Pluronic F-127 (Life Technologies):50% DMSO) in 5 mL saline solution (140 mM NaCl, 5 mM KCl, 1 mM MgCl_2_, 2 mM CaCl_2_, 10 mM glucose and 10 mM HEPES, adjusted to pH 7.4). Plates were incubated for 60 min at 37 °C, 5% CO_2_. Excess dye solution was removed, and fresh saline solution added and baseline florescence readings (fura-2 AM = excitation 340 nm/emission 520 nm, fluo-4 AM = excitation 480 nm/emission 520 nm) were taken for 1 s using a FlexStation 3 (Molecular Devices, San Jose, CA, USA). The automatic pipetting system on the FLEX station was set to add the taste compound after 30 s of plate reading and readings were taken every 5 s for 1 min following compound addition. Ionomycin (10 μM) was added by the FLEX station, as a control to elicit maximum response in cells and allow for normalisation between wells, after 1 min and readings continued for 30 s more. Data were analysed using Softmax Pro software and expressed as the ratio between excitation and emission spectra (fura-2) or the difference between maximum florescence and baseline florescence (expressed as a ratio of ionomycin florescence) (fluo-4). 

For measurement of responses to caffeine, probenecid (Sigma, Dorset, UK) (1 mM final dilution, 2 µL of 500 mM stock, prepared in 1N NaOH added per 1 mL saline) was added to the fluorescent dye/saline mixture and used to prevent leakage of calcium indicator dye. However, due to the reported allosteric inhibitory action of probenecid on TAS2R38 receptors [33], it was not used in PTC experiments except as an inhibitor to control for TAS2R38 independent responses. Some dye leakage did occur in cells where probenecid was not added and as a result, the florescence readings were lower in those experiments.

## 3. Statistics

A power calculation (G*Power Software, Düsseldorf, Germany) was used to determine the sample size based on an average (±SD) difference in salivary flow rate and mucin concentration between older and younger adults. Assuming a medium effect size (d = 1.12, d = 1.09 respectively) a sample of *n* = 20 would be required to achieve significance (*p* < 0.05) using an unpaired *t* test (95% statistical power) [34]. Microsoft Excel (Version 1804, Microsoft Corporation, Redmond, WA, USA) and GraphPad Prism 7 software (GraphPad Software Inc., La Jolla, CA, USA) was used for data analysis and generation of graphs. SPSS version 24 was also used for statistical analysis (IBM Analytics, Armonk, NY, USA). The data was tested for normal distribution using the D’Agostino and Pearson’s normality test. Data which was not normally distributed was analysed using non-parametric tests (Friedman test with Dunn’s multiple comparisons). Data which was normally distributed was analysed with parametric tests (ANOVA). The student’s *t* test was selected in the case of testing difference between two variables, even in the case of abnormal distribution when there was a great enough ‘n’ number to allow use of the parametric test [35]. Significance = *p* value < 0.05 * *p* < 0.01 ** *p* < 0.001 ***, *p* < 0.0001 ****. 

While the sample collection and analysis were conducted by the same person, all samples were pseudo-anonymised with a code which was only broken after the analyses was complete, in order to mitigate the effects of researcher bias.

## 4. Results

### 4.1. Comparative Viscosity of Saliva in Ageing

**Figure 1:**
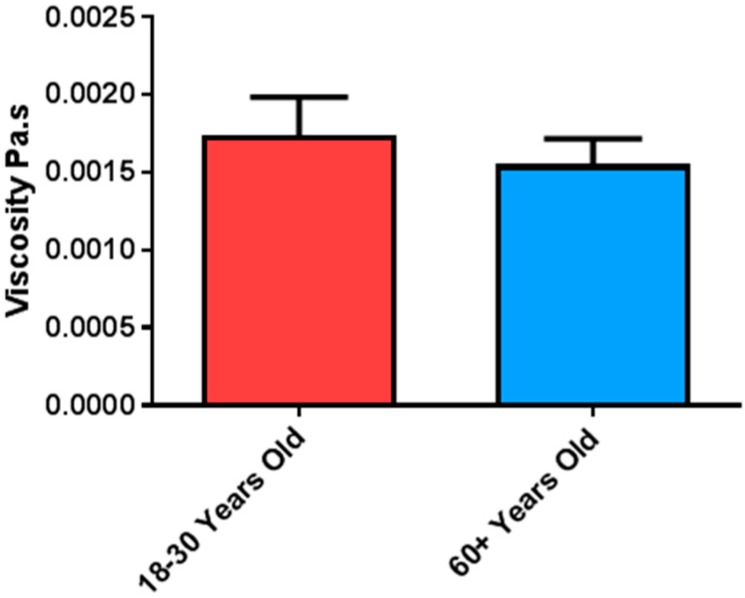
Mean (+/−SEM) dynamic viscosity of unstimulated whole mouth saliva (UWS) from older (*n* = 24) and younger (*n* = 30) subjects, determined using dynamic differential microscopy (DDM).

Viscosity of unstimulated whole mouth saliva (UWS), from both age groups, was measured to determine the effect of ageing on bulk rheology of saliva. The dynamic viscosity of UWS was not significantly different between the age groups. However, the younger group did have slightly higher viscosity, 1.73 +/− 0.2 mPa·s compared to 1.55 +/− 0.2 mPa·s in the older group (Figure 1).

### 4.2. Analysis of MUC5b and MUC7 Expression in Saliva from Older and Younger Adults

**Figure 2:**
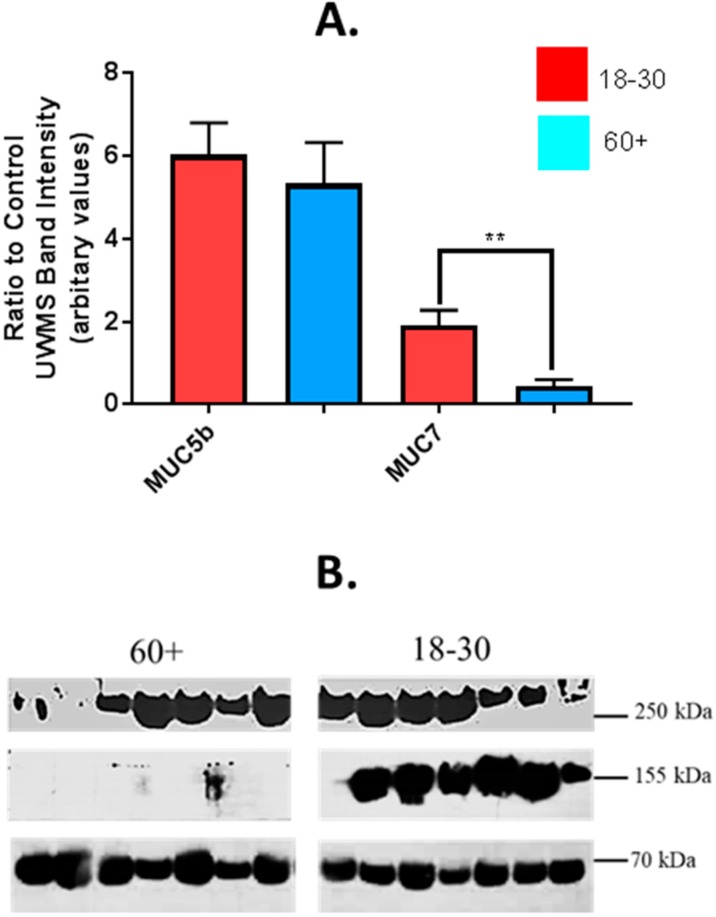
MUC5b and MUC7 in UWS of older and younger adults determined by immuno-blotting (**A**) Mean (+/−SEM) MUC5b and MUC7 expression in UWS from older (*n* = 24) and younger (*n* = 30) subjects, determined by immuno-blotting. Band intensity expressed as a ratio to band intensity of control UWS sample. Difference in means between groups tested for statistical significance using student’s *t* test. *p* = < 0.05 * *p* = < 0.01 ** *p* = < 0.001 ***. (**B**) Representative image of an SDS-PAGE gel PAS of UWS from older (60+ years) and younger (18–30 years) adults stained for mucin glycoproteins. Approximate molecular weights: MUC5b 250 kDa, MUC7 155 kDa, amylase (used as loading control) 55 kDa.

Previous studies have shown reduced MUC7 in saliva from older adults, using glycoprotein staining of SDS PAGE gel electrophoresis of saliva [34,36]. Glycoprotein staining, such as PAS and stains-all used in previous studies, show glycosylated forms of mucin, therefore de-glycosylation of mucin due to degradation, would show as reduced levels when quantifying using this method [37]. To determine whether expression of MUC7 is reduced or if MUC7 is de-glycosylated in UWS of older adults, immuno-blotting of mucins was conducted with anti-bodies directed to the peptide core of MUC7 and MUC5b as previously described [38,39]. Mean (+/−SEM) levels of MUC7 were significantly higher in UWS from younger adults compared to older, 1.90 +/− 0.37 and 0.453 +/− 0.15 respectively, *p* = < 001 (Figure 2). There was no significant difference in MUC5b from UWS of either age group, when semi-quantified from immuno-blotting, mean ratio to control WS of 6.02 +/− 0.77 in the younger group and 5.328 +/− 0.99 in the older group (Figure 2). 

### 4.3. Effect of Age on Sialylation of MUC5b and MUC7 

**Figure 3:**
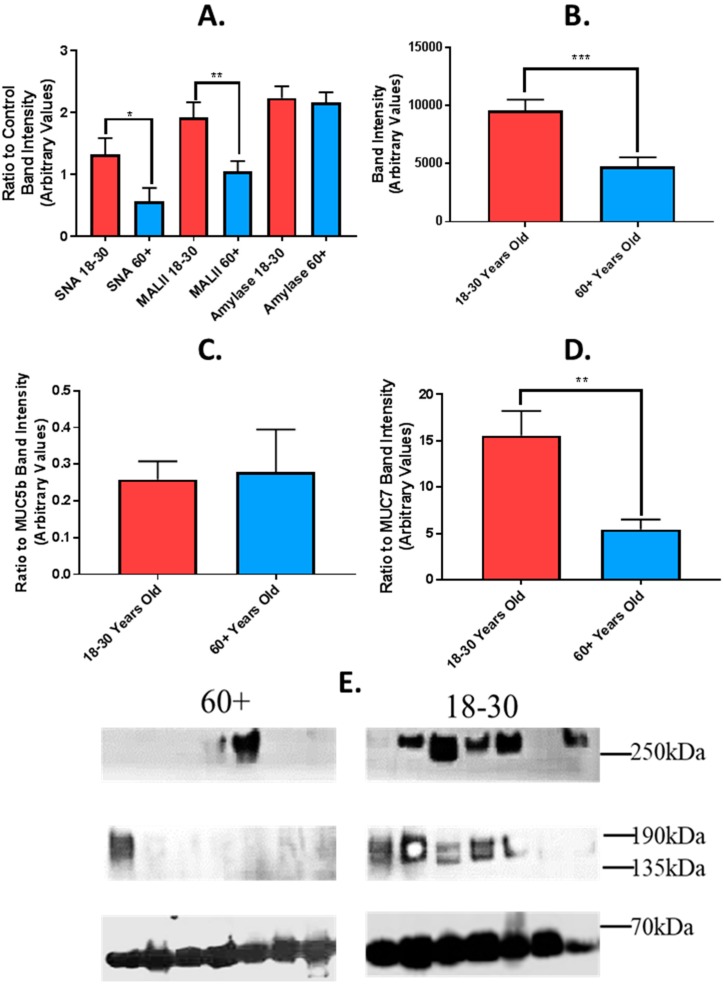
Sambucus Nigra Lectin (SNA) and Maackia amurensis lectin II (MALII) lectin binding in UWS from older and younger adults. (**A**) Mean (+/−SEM) SNA and MAL II lectin, respectively, binding in UWS from older (*n* = 22) and younger subjects (*n* = 30). Mean (+/−SEM) amylase in UWS, used as loading control. (**B**) Mean (+/−SEM) total sialic acid levels in UWS from older and younger subjects. Calculated as total band intensity of SNA + total band intensity of MAL II. (**C**) Ratio of SNA lectin to total MUC5b (determined by immuno-blot) in UWS of older and younger subjects. (**D**) Ratio of MALii lectin to total MUC7 (determined by immuno-blot) in UWS of older and younger subjects. Difference in means between groups tested for statistical significance using student’s *t* test. *p* = < 0.05 * *p* = < 0.01 ** *p* = < 0.001 ***. (**E**) Representative image of lectin Western blots. Detection of sialic acids in UWS of younger (18–30) and older (60+) adults. SNA > 250 kDa, MALII 155 kDa, Amylase 55 kDa.

To further investigate mucin degradation in UWS from older and younger adults, lectin immuno-blotting was conducted with SNA and MAL II to detect changes in sialylation. SNA binds preferentially to MUC5b while MAL II binds preferentially to MUC7 [38]. There were significantly higher levels of both SNA and MAL II binding to mucins in UWS of younger adults compared to older (Figure 3A). For SNA, the mean (+/−SEM) ratio to the control (amylase) in UWS was 1.33 +/− 0.22 in younger adults and 0.56 +/− 0.26 in older adults, *p* = < 0.05. For MAL II, the mean (+/−SEM) ratio to the control UWS was 1.92 +/− 0.17 in younger adults and 1.046 +/− 0.2498 in older adults, *p* = <0.01. Levels of salivary amylase did not differ significantly between groups (Figure 3A). Band intensities from probing with both lectins were combined to give a value for total sialic acid in UWS from both age groups (Figure 3B). The level of total sialic acid was significantly higher in UWS of younger adults, with a mean (+/−) band intensity of 9565 +/− 939.5 compared to 4741 +/− 816.3 in the older group (arbitrary values), *p* = < 0.001. When expressed as a ratio to total mucin expression, the ratio of SNA binding to MUC5b did not differ between groups (Figure 3C). The ratio of MAL II binding to MUC7 was significantly higher in the younger group, 15.57 +/− 1.08 compared to 5.45 +/− 2.64 in the older group, *p* = < 0.01 (Figure 3D). 

Thus, although there is no significant difference in levels of MUC5b between age groups, levels of sialylation are significantly decreased in the older group. In contrast, the lower binding of SNA to MUC7 may reflect both reduced levels of the mucin and reduced sialylation in the older age group. 

### 4.4. Effect of Age of Donor on Salivary MUC5b Binding in an In Vitro Model of the Oral Epithelium 

**Figure 4:**
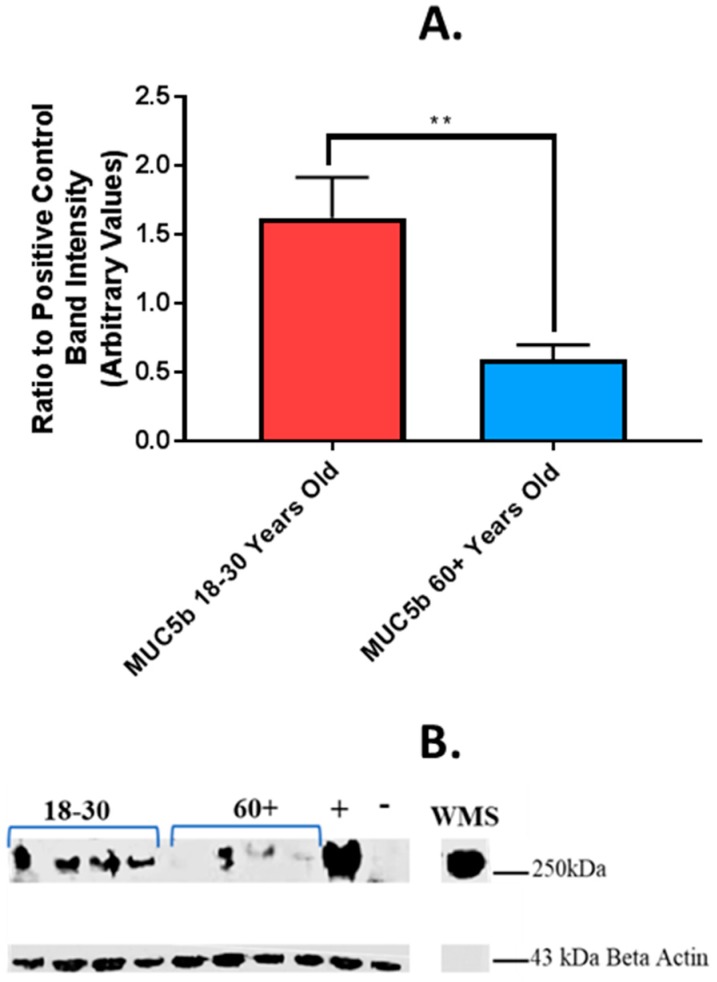
Binding of MUC5b from UWS of older and younger adults to oral epithelial cells. (**A**) Mean (+/−SEM) binding of MUC5b from UWS of older (*n* = 21) and younger (*n* = 29) adults to TR146/MUC1 cells. MUC5b was quantified by blotting with anti-MUC5b antibody. (**B**) Representative image of immuno-blot for MUC5b (>250 kDa) from UWS of older and younger adults, binding to TR146/MUC1 cells. + = binding of control UWS sample, − = negative control (no WS), WS = control WS sample run as a comparison. Beta actin (43 kDa) used as loading control. Difference in means between groups tested for statistical significance using student’s *t* test. *p* = < 0.05 * *p* = < 0.01 **.

Mucins facilitate binding of the salivary pellicle to the tongue and may therefore promote the access of bitter tastants to the taste receptors. To optimise the in vitro model for muco-adhesion of saliva, control UWS from one healthy donor was used on SCC090 and TR146/MUC1 cells (Figure S1). Cells were incubated with saliva for 2 h and then washed to remove un-bound saliva and lysed. The residual, bound, saliva was determined by immuno-blotting of cell lysates. Both cell lines exhibited binding to salivary MUC5b (Figure S1). Bound MUC7 was not detected in either cell line (Figure S1B). MUC1 expression was evident in both cell lines (Figure S1B). Binding of MUC5b in UWS from both age groups was determined in the TR146/MUC1 cell model. There was significantly higher binding of MUC5b from UWS of younger adults compared to older, 1.62 +/− 0.30 and 0.60 +/− 0.10 respectively, *p* = < 0.01 (Figure 4). 

### 4.5. Modulation of In Vitro Responses to Tastants by Saliva from Older and Younger Adults

**Figure 5:**
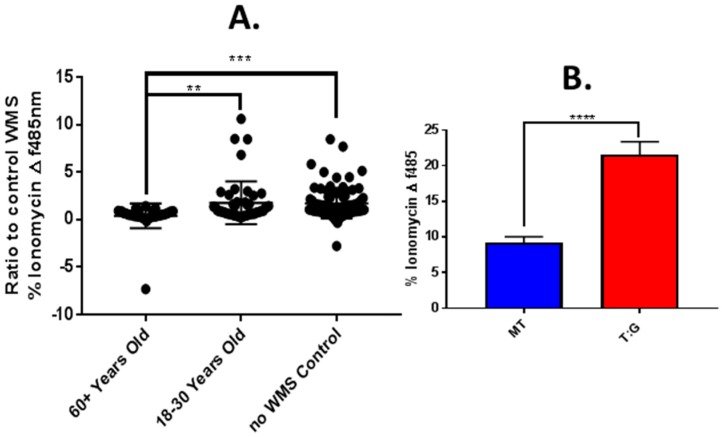
FLEX station measurement of intracellular calcium with PTC in TR146 MUC1 cells with and without UWS from older and younger adults. TR146 MUC1 Cells were loaded with the fluo-4am calcium indicator and incubated with or without UWS from older (60+ years, *n* = 22) and younger (18–30 years, *n* = 29) adults. Florescence emissions were recorded using FLEX station fluorescence plate reader, before and after addition of 25 µM TC. (**A**) Mean (+/−SEM) change (∆) in fluorescence from baseline after compound addition (normalised to ionomycin response and control WS response), in TAS2R38:Ga16Gust44 co-transfected TR146 MUC1 cells. Cells stimulated with 25 µM PTC without WS used for comparison (*n* = 44). Data is representative of two experiments. (**B**) Transfection efficiency of TR146 MUC1 cells, without whole mouth saliva (WS). Mean (+/−SEM) change (∆) in fluorescence from baseline after compound addition, in mock transfected (MT) and TAS2R38:Ga16Gust44 co-transfected (T:G) TR146 MUC1 cells (*n* = 20). Analysed for statistical significance using independent student’s *t* test. Significance = *p* value < 0.05 * *p* < 0.01 ** *p* < 0.001 ***.

The effect of age-related changes in UWS on calcium responses to PTC were demonstrated by incubating TAS2R38:Gα16Gust44 transfected TR146 MUC1 cells with UWS from older and younger adults. Expression of TAS2R38 and Gα16Gust44 were confirmed using RT-PCR and Western blotting (Figures S2, S4, S5 and S7). Transfection conditions and were also optimised (Figure S3). PTC at 25µM was selected for the experiment since it was the lowest concentration required to elicit a TAS2R38 specific response, i.e., a significantly greater response in TAS2R38:Gα16Gust44 transfected cells compared to mock transfected (Figure S8). A significantly reduced response to PTC was observed in the presence of UWS from older adults (*n* = 22) with a mean (+/−SEM) florescence value of 0.39 +/− 0.21 compared to 1.76 +/− 0.33 with UWS from younger adults (*n* = 29) and 1.71 +/− 0.17 (*p* = < 0.01) without UWS (*p* = < 0.001) (arbitrary values) (Figure 5A). There was no significant difference between responses with younger UWS and responses without UWS. Successful transfection was demonstrated, since the response to PTC was significantly greater in TAS2R38:Gα16Gust44 transfected cells, 21.57 +/− 0.81, compared to the response in mock transfected cells which was 9.22 +/−1.80 (*p* = < 0.0001) (Figure 5B).

**Figure 6:**
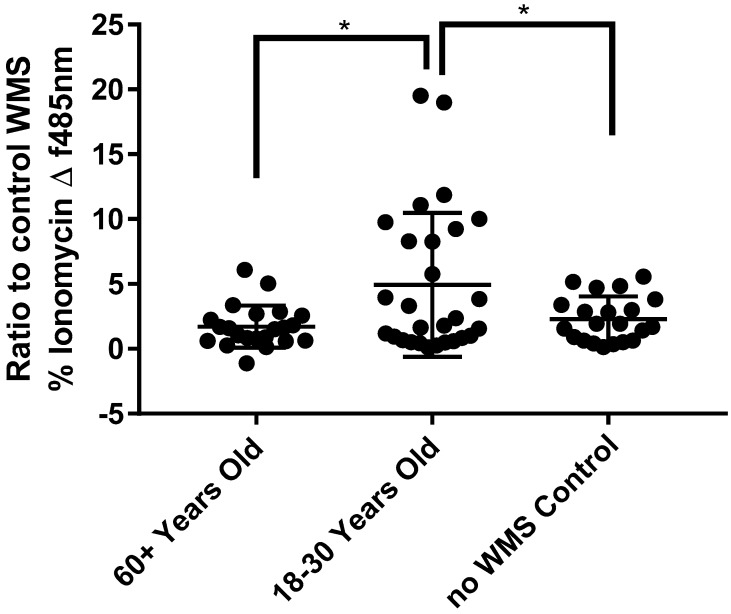
FLEX station measurement of intracellular calcium with caffeine in SCC090 cells with and without UWS from older and younger adults. SCC090 cells were loaded with the fluo-4am calcium indicator and incubated with or without UWS from older (60 + years, *n* = 22) and younger (18–30 years, *n* = 28) adults. Fluorescence emissions were recorded using FLEX station fluorescence plate reader, before and after addition of 0.16 mM caffeine. Mean (+/−SEM) change (∆) in fluorescence from baseline after compound addition (normalised to ionomycin response and control WS response). Cells stimulated with 0.16 mM caffeine without WS used for comparison (*n* = 21). Analysed for statistical significance using independent student’s *t* test. Significance = *p* < 0.05 * *p* < 0.01 ** *p* < 0.001 ***.

To demonstrate the effect of age-related changes in UWS on taste receptor interactions with caffeine, intracellular calcium responses in SCC090 cells were measured in the presence of UWS from older and younger adults. Caffeine concentrations were chosen for this experiment based on calcium response in SCC090 cells during optimisation experiments (Figure S9). A significantly higher response to 1.6 mM caffeine was evident in the presence of UWS from younger adults, with a mean florescence value (+/−SEM) of 4.94 +/− 1.05, compared with UWS from older adults, 1.72 +/−0.35 (*p* = < 0.05) and without saliva, 2.3 +/− 0.38 (*p* = < 0.05) (arbitrary values) (Figure 6). The response in the presence of UWS from older adults was not significantly different to the response seen without UWS (Figure 6). 

## 5. Discussion

Our previous study with this cohort of participants indicated that the older age group perceived certain stimuli (monosodium glutamate, PTC, and menthol odour) less strongly than the younger group [7]. When salivary flows were measured in response to the different tastants the older group generally showed a lower flow rate suggesting that subjective effects alone were not responsible for these differences. Although the older group took more medication than the younger group, their resting salivary flow rate was >0.1 g/min—above the range for xerostomia and we considered the older group healthy. One observation requiring further examination was the greatly reduced spinnbarkeit of the older group’s saliva compared to the younger group. Since the spinnbarkeit is closely related to the formation of the mucosal pellicle the present study was an in-depth analysis of the salivary mucins in both groups to determine if differences in the functionality could affect taste processes, in particular the transfer of tastants to taste receptors.

Since muco-adhesion is an important functional property of salivary mucin which may affect taste function, a muco-adhesion assay was performed to assess the effect of altered mucin sialylation and glycosylation on adhesive capabilities of saliva in older adults. TR146 cells which stably express MUC1 were selected as a model for the oral mucosa as surface bound MUC1 facilitates mucin-mucin binding to MUC5b [31]. In the present study, the adherence of UWS MUC5b from older adults was reduced compared to younger adults. Despite the total levels of MUC5b being similar between age groups, the functionality of this mucin and binding to the oral epithelium may be impaired in older adults. The age-related reduction in salivary muco-adhesion seen in this study could be an underestimate of that occurring in vivo, since MUC1 expression has also been shown to be reduced in the oral epithelium of older adults which may further reduce muco-adhesion, in vivo [40].

In vitro models for taste receptor activation are widely used to study the interaction between taste compounds and receptors [13,14,15]. However, previous studies have used cell lines from extra oral locations which may not be representative of the tongue epithelium. Moreover, the role played by saliva, in tastant–receptor interaction has not been considered. In this study, a biologically relevant model for taste receptor activation was developed, using oral epithelial cells. Calcium responses to PTC in TAS2R38 transfected TR146 cells were significantly reduced in the presence of UWS from older adults, compared with responses in buffer alone while responses with UWS from younger adults were similar to buffer. This suggests that the transport of PTC to the TAS2R38 receptor may be reduced in the older age group. Similarly, in the SCC090 cell line model, calcium responses to caffeine were enhanced by the presence of UWS from younger adults compared to UWS from older adults and buffer control. 

If the layer of saliva on the tongue is reduced, because it is easily washed away and not bound to the mucosa through MUC1/MUC5b interactions, the dissolution of tastants may be impaired. Increased surface binding of saliva may lead to the enhanced retention of taste compounds in the oral cavity, which could perpetuate responses [41]. Lipophilic and hydrophobic compounds, for example, bitter tastants such as caffeine and PTC, bind to mucin in saliva [42,43,44]. Therefore, highly muco-adhesive saliva may facilitate the retention of these tastants in the mouth, allowing them to remain available to the taste receptors for a longer period. Indeed, older adults generally display reduced responsiveness to lyophilic taste compounds compared to younger adults [45].

As an amphiphile, caffeine can increase the solubility of hydrophobic molecules in water [46,47] as well as preventing the aggregation of such molecules. [48]. By binding to mucins, caffeine may inhibit their aggregation and thus promote faster diffusion to reach the taste receptors. In older adults, who displayed reduced salivary mucin and reduced sialylation of mucin with associated reduced functionality (i.e., reduced viscoelasticity of saliva) these mechanisms may not operate. 

Expression of TAS2R10 and TAS2R43 were demonstrated in SCC090 cells (see Figure S6) but there are multiple other TAS2Rs that were not investigated which could be responsible for caffeine responses. Furthermore, multiple non-taste receptors exist which may also be activated by caffeine leading to intracellular calcium release. The lack of specific inhibitors of TAS2R receptors meant it was not possible to clearly demonstrate which receptor is activated during caffeine stimulation of SCC090 cells. 

To assess the changes in quality of saliva in ageing, the viscosity of UWS samples from older and younger adults was measured using DDM, a useful tool for measuring the viscosity of low volume samples. The salivary viscosity values determined in this study are in agreement with several previous studies [49,50,51,52] although, others have reported higher values [53,54,55]. Variation between studies likely reflects the lack of standardisation in testing methods and parameters such as temperature and shear rate. 

There was no significant difference in UWS viscosity between age groups which corroborates previous work by Briedis et al., who showed no difference in SWS viscosity between older and younger adults [56]. Generally, the viscosity of saliva was higher in the younger group however this may reflect the fact that viscoelasticity was also greater in UWS of younger adults. DDM may be affected by viscoelasticity of the sample since movement of the colloid particles may be different in an elastic fluid compared to a purely viscous sample, known as Brownian motion [57]. However, the effect of viscoelasticity should be minimal in this study, as samples were frozen and thawed before the measurement [58,59].

The lower levels of MUC7 in older adults compared to younger may be the result of proteolysis of the peptide core or reduced secretion of mucin in older adults. Mucin degradation is sequential and begins with de-sialylation, followed by the degradation of oligosaccharide side chains, which exposes the protein core to proteolytic enzymes [60]. To further investigate the effect of ageing on the degradation of salivary mucin, lectin immuno-blots were conducted using SNA and MAL II to detect α-2, 3 and α-2, 6 linked sialic acid residues. Levels of both sialic acids were lower in older adults compared to younger. MUC7 sialylation was still reduced even when accounting for the reduced total levels, confirming degradation in saliva of older adults. Reduced sialylation of MUC7 has also been demonstrated in Sjögren’s syndrome patients and may be associated with decreased lubrication and protection of the oral mucosa [38]. The degradation of mucin glycans and the reduction in sialylation results in a decrease in viscoelasticity of saliva with reduced hydration [61,62].

The use of medication amongst volunteers may represent a limitation of this study, since certain medications are known to affect salivary flow and taste. However, the study group may be better representative of the older population, since the vast majority of older adults in the UK take one or more medication [63]. 

## 6. Conclusions

The results from this study demonstrate a decrease in the quantity and glycosylation of salivary mucins in older adults. De-sialylation and de-glycosylation of MUC7 in saliva of older adults affect mucin function. This is the first study to suggest that altered muco-adhesiveness of saliva may be an important factor in taste function which has not until now been considered. But how might impaired binding of MUC5b from UWS to TR146/MUC1 cells cause the reduced taste transduction demonstrated by the transfected-TR146 and SCC090 cell lines? One possible mechanism is that the adhered mucins provide an extracellular matrix than helps to concentrate tastants near to taste receptors. This could presumably explain the calcium responses to caffeine which were no higher than the buffer control when tastant was added in the presence of saliva from older adults but were significantly enhanced in the presence of saliva from younger adults. However, for more hydrophilic tastants like salt and sugar, this mechanism seems unlikely unless mucin hydrogels were able to bind sodium and saccharides, which has not been demonstrated so far. An obvious limitation of this work is the use of single tastants in aqueous solutions rather than real foods. Further work is therefore required using real foods to explore their interaction with salivary mucins in solution and when adhered to the mucosa (the mucosal pellicle). To conclude, we suggest that reduced taste sensation in older adults may, at least in part, reflect a decrease in mucin and mucin glycosylation which in turn leads to reduced muco-adhesion and impaired tastant–receptor interactions. 

## Figures and Tables

**Table 1:** Oligonucleotides used.

Oligo Name	5′–3′ Sequence	Expected Product Size (Base Pairs)	Melting Temperature (°C)
**Gα16Gust44 forward**	CCT GGT TCA AAA GCA CAT CCG	254	68.6
**Gα16Gust44 reverse**	TTG GGT GTC AGT AGC ACA GGT		64.6
**YWHAZ forward**	ACT TTT GGT ACA TTG TGG CTT CAA	94	65.3
**YWHAZ reverse**	CCG CCA GGA CAA ACC AGT AT		66.0
**TAS2R7 forward**	GGA TTC TAC TGG GGT GCG TGG T	260	70.8
**TAS2R7 reverse**	ATA GTC CGC TTA CGT CGA GTC AC		65.4
**TAS2R43 forward**	ATC TGG GCA GTG ATC AAC CA	156	65.6
**TAS2R43 reverse**	TAG CAA AGG CCC CAA CAA CA		67.5
**TAS2R38 forward**	AGG CCC ACA TTA AAG CCC TC	204	60.03
**TAS2R38 reverse**	CAG CTC TCC TCA ACT TGG CA		59.96
**TAS2R10 forward**	GTG TAG TGG AAG GCA TCT TCA	296	61.7
**TAS2R10 reverse**	GCT GGT GGC AAA CCA CAT AC		65.3

## Supplementary Materials

The following are available online at https://www.mdpi.com/2072-6643/11/10/2280/s1, Figure S1: Binding of MUC5b from control UWS to oral epithelial cells. Figure S2: RT-PCR amplification of mRNA from TR146 MUC1 cells, transiently transfected with cDNA encoding for hTAS2R38 and the G protein chimera Ga16Gust44. Figure S3: Increasing concentrations of TAS2R38 and Ga16Gust44 cDNA and varying ratios of TAS2R38:Ga16Gust44 were used to transiently transfect/co-transfect cells. Figure S4: TR146 cells were transiently transfected with three increasing concentrations of TAS2R38 and the chimeric FLAG tagged Gα16Gust44 cDNA and three varying ratios of TAS2R38:Gα16Gust44 cDNA. Figure S5: Representative immuno-blot image of TAS2R38 in transfected TR146 (A.) and TR146 MUC1 (B.) cells shown without (−) and with (+) immunising peptide added to the primary antibody to block non-specific binding. Figure S6: mRNA from SCC090 cells were used for RT-PCR of TAS2R bitter receptors. Figure S7: Immunising peptide against TAS2R38 was used to determine antibody specificity., Figure S8: Intracellular calcium changes in transfected TR146 MUC1 cells loaded with the fura2-am calcium indicator with increasing concentrations of PTC. Figure S9: SCC090 cells were loaded with the fura2-am calcium indicator and florescence emissions recorded before and after exposure of the cells to increasing concentrations of bitter receptor agonists.

## Abbreviations

BCA	Bicinchoninic acid
MUC1	mucin type 1
MUC5b	mucin type 5, b
MUC7	mucin type 7
T1R/TAS1R	taste receptor type 1
T2R/TAS2R	taste receptor type 2
WS	whole mouth saliva
UWS	un-stimulated whole mouth saliva
SWS	stimulated whole mouth saliva

## References

[1] Yoshinaka M., Ikebe K., Uota M., Ogawa T., Okada T., Inomata C., Takeshita H., Mihara Y., Gondo Y., Masui Y. (2016). Age and sex differences in the taste sensitivity of young adult, young-old and old-old Japanese. Geriatr. Gerontol. Int..

[2] Methven L., Allen V.J., Withers C.A., Gosney M.A. (2012). Ageing and taste. Proc. Nutr. Soc..

[3] Prince M.J., Wu F., Guo Y., Robledo L.M.G., O’Donnell M., Sullivan R., Yusuf S. (2015). The burden of disease in older people and implications for health policy and practice. Lancet.

[4] Brownie S. (2006). Why are elderly individuals at risk of nutritional deficiency?. Int. J. Nurs. Pract..

[5] Matsuo R. (2000). Role of Saliva in the Maintenance of Taste Sensitivity. Crit. Rev. Oral Biol. Med..

[6] Mese H., Matsuo R. (2007). Salivary secretion, taste and hyposalivation. J. Oral Rehabil..

[7] Pushpass R.-A.G., Daly B., Kelly C., Proctor G., Carpenter G.H. (2019). Altered salivary flow, protein composition and rheology following taste and TRP stimulation in older adults. Front. Physiol..

[8] Cook S.L., Bull S.P., Methven L., Parker J.K., Khutoryanskiy V.V. (2017). Mucoadhesion: A food perspective. Food Hydrocoll..

[9] Gallardo-Escamilla F., Kelly A., Delahunty C. (2007). Mouthfeel and flavour of fermented whey with added hydrocolloids. Int. Dairy J..

[10] Cook S.L., Woods S., Methven L., Parker J.K., Khutoryanskiy V.V. (2018). Mucoadhesive polysaccharides modulate sodium retention, release and taste perception. Food Chem..

[11] Gardner R. (1978). Lipophilicity and bitter taste. J. Pharm. Pharmacol..

[12] Wiet S., Miller G. (1997). Does chemical modification of tastants merely enhance their intrinsic taste qualities?. Food Chem..

[13] Brockhoff A., Behrens M., Massarotti A., Appendino G., Meyerhof W. (2007). Broad tuning of the human bitter taste receptor hTAS2R46 to various sesquiterpene lactones, clerodane and labdane diterpenoids, strychnine, and denatonium. J. Agric. Food Chem..

[14] Bufe B., Hofmann T., Krautwurst D., Raguse J.-D., Meyerhof W. (2002). The human TAS2R16 receptor mediates bitter taste in response to β-glucopyranosides. Nat. Genet..

[15] Meyerhof W., Batram C., Kuhn C., Brockhoff A., Chudoba E., Bufe B., Appendino G., Behrens M. (2010). The molecular receptive ranges of human TAS2R bitter taste receptors. Chem. Senses.

[16] Hershkovich O., Nagler R.M. (2004). Biochemical analysis of saliva and taste acuity evaluation in patients with burning mouth syndrome, xerostomia and/or gustatory disturbances. Arch. Oral Biol..

[17] Temmel A.F., Quint C., Schickinger-Fischer B., Hummel T. (2005). Taste function in xerostomia before and after treatment with a saliva substitute containing carboxymethylcellulose. J. Otolaryngol..

[18] Satoh-Kuriwada S., Shoji N., Kawai M., Uneyama H., Kaneta N., Sasano T. (2009). Hyposalivation strongly influences hypogeusia in the elderly. J. Health Sci..

[19] Matsuo R., Yamauchi Y., Morimoto T. (1997). Role of submandibular and sublingual saliva in maintenance of taste sensitivity recorded in the chorda tympani of rats. J. Physiol..

[20] Nanda R., Catalanotto F.A. (1981). Basic biological sciences: Long-term effects of surgical desalivation upon taste acuity, fluid intake, and taste buds in the rat. J. Dent. Res..

[21] Gurkan S., Bradley R.M. (1987). Autonomic control of von Ebner’s lingual salivary glands and implications for taste sensation. Brain Res..

[22] Gurkan S., Bradley R.M. (1988). Secretions of von Ebner’s glands influence responses from taste buds in rat circumvallate papilla. Chem. Senses.

[23] De Almeida Pdel V., Gregio A.M., Machado M.A., de Lima A.A., Azevedo L.R. (2008). Saliva composition and functions: A comprehensive review. J. Contemp. Dent. Pract..

[24] Pedersen A.M.L., Sørensen C.E., Proctor G., Carpenter G., Ekström J. (2018). Salivary secretion in health and disease. J. Oral Rehabil..

[25] DeSimone J.A., Heck G.L. (1980). An analysis of the effects of stimulus transport and membrane charge on the salt, acid and water-response of mammals. Chem. Senses.

[26] Bufe B., Breslin P.A., Kuhn C., Reed D.R., Tharp C.D., Slack J.P., Kim U.-K., Drayna D., Meyerhof W. (2005). The molecular basis of individual differences in phenylthiocarbamide and propylthiouracil bitterness perception. Curr. Biol..

[27] Kim U., Drayna D. (2005). Genetics of individual differences in bitter taste perception: Lessons from the PTC gene. Clin. Genet..

[28] Kim A.J., Manoharan V.N., Crocker J.C. (2005). Swelling-based method for preparing stable, functionalized polymer colloids. J. Am. Chem. Soc..

[29] Cerbino R., Cicuta P. (2017). Perspective: Differential dynamic microscopy extracts multi-scale activity in complex fluids and biological systems. J. Chem. Phys..

[30] Feriani L., Juenet M., Fowler C.J., Bruot N., Chioccioli M., Holland S.M., Bryant C.E., Cicuta P. (2017). Assessing the Collective Dynamics of Motile Cilia in Cultures of Human Airway Cells by Multiscale DDM. Biophys. J..

[31] Ployon S., Belloir C., Bonnotte A., Lherminier J., Canon F., Morzel M. (2016). The membrane-associated MUC1 improves adhesion of salivary MUC5B on buccal cells. Application to development of an in vitro cellular model of oral epithelium. Arch. Oral Biol..

[32] Ueda T., Ugawa S., Yamamura H., Imaizumi Y., Shimada S. (2003). Functional interaction between T2R taste receptors and G-protein α subunits expressed in taste receptor cells. J. Neurosci..

[33] Greene T.A., Alarcon S., Thomas A., Berdougo E., Doranz B.J., Breslin P.A., Rucker J.B. (2011). Probenecid inhibits the human bitter taste receptor TAS2R16 and suppresses bitter perception of salicin. PLoS ONE.

[34] Navazesh M., Mulligan R.A., Kipnis V., Denny P.A., Denny P.C. (1992). Comparison of whole saliva flow rates and mucin concentrations in healthy Caucasian young and aged adults. J. Dent. Res..

[35] Lumley T., Diehr P., Emerson S., Chen L. (2002). The importance of the normality assumption in large public health data sets. Annu. Rev. Public Health.

[36] Denny P.C., Denny P.A., Klauser D.K., Hong S.H., Navazesh M., Tabak L.A. (1991). Age-related changes in mucins from human whole saliva. J. Dent. Res..

[37] Baughan L., Robertello F., Sarrett D., Denny P., Denny P. (2000). Salivary mucin as related to oral Streptococcus mutans in elderly people. Mol. Oral Microbiol..

[38] Chaudhury N.M.A., Proctor G.B., Karlsson N.G., Carpenter G.H., Flowers S.A. (2016). Reduced Mucin-7 (Muc7) Sialylation and Altered Saliva Rheology in Sjögren’s Syndrome Associated Oral Dryness. Mol. Cell. Proteom..

[39] Rousseau K., Wickstrom C., Whitehouse D.B., Carlstedt I., Swallow D.M. (2003). New monoclonal antibodies to non-glycosylated domains of the secreted mucins MUC5B and MUC7. Hybrid. Hybridomics.

[40] Chang W.-I., Chang J.-Y., Kim Y.-Y., Lee G., Kho H.-S. (2011). MUC1 expression in the oral mucosal epithelial cells of the elderly. Arch. Oral Biol..

[41] Malkki Y., Heinio R., Autio K. (1993). Influence of oat gum, guar gum and carboxymethyl cellulose on the perception of sweetness and flavor. Food Hydrocoll..

[42] Larhed A.W., Artursson P., Gråsjö J., Björk E. (1997). Diffusion of drugs in native and purified gastrointestinal mucus. J. Pharm. Sci..

[43] Norris D.A., Sinko P.J. (1997). Effect of size, surface charge, and hydrophobicity on the translocation of polystyrene microspheres through gastrointestinal mucin. J. Appl. Polym. Sci..

[44] Matthes I., Nimmerfall F., Sucker H. (1992). Mucus models for investigation of intestinal absorption mechanisms. 2. Mechanisms of drug interactions with intestinal mucus. Die Pharm..

[45] Schiffman S.S., Gatlin L.A., Frey A.E., Heiman S.A., Stagner W.C., Cooper D.C. (1994). Taste perception of bitter compounds in young and elderly persons: Relation to lipophilicity of bitter compounds. Neurobiol. Aging.

[46] Lim L.-Y., Go M.-L. (2000). Caffeine and nicotinamide enhances the aqueous solubility of the antimalarial agent halofantrine. Eur. J. Pharm. Sci..

[47] Rasool A.A., Hussain A.A., Dittert L.W. (1991). Solubility enhancement of some water-insoluble drugs in the presence of nicotinamide and related compounds. J. Pharm. Sci..

[48] Sharma B., Paul S. (2016). Role of caffeine as an inhibitor in aggregation of hydrophobic molecules: A molecular dynamics simulation study. J. Mol. Liq..

[49] Van der Reijden W., Veerman E., Nieuw Amerongen A. (1993). Shear rate dependent viscoelastic behavior of human glandular salivas. Biorheology.

[50] Van der Reijden W.A., Veerman E.C., Nieuw Amerongen A.V. (1994). Rheological properties of commercially available polysaccharides with potential use in saliva substitutes. Biorheology.

[51] Sajewicz E. (2009). Effect of saliva viscosity on tribological behaviour of tooth enamel. Tribol. Int..

[52] Sánchez E.R.B., Honores M.J.C. (2015). Effect of orthodontic fixed appliances on salivary flow and viscosity. Rev. Mex. Ortod..

[53] Rantonen P.J., Meurman J.H. (1998). Viscosity of whole saliva. Acta Odontol. Scand..

[54] Gittings S., Turnbull N., Henry B., Roberts C.J., Gershkovich P. (2015). Characterisation of human saliva as a platform for oral dissolution medium development. Eur. J. Pharm. Biopharm..

[55] Ligtenberg A., Liem E., Brand H., Veerman E. (2016). The effect of exercise on salivary viscosity. Diagnostics.

[56] Briedis D., Moutrie M., Balmer R. (1980). A study of the shear viscosity of human whole saliva. Rheol. Acta.

[57] Martin D.S., Forstner M.B., Käs J.A. (2002). Apparent subdiffusion inherent to single particle tracking. Biophys. J..

[58] Jaedicke K.M., Taylor J.J., Preshaw P.M. (2012). Validation and quality control of ELISAs for the use with human saliva samples. J. Immunol. Methods.

[59] Schipper R.G., Silletti E., Vingerhoeds M.H. (2007). Saliva as research material: Biochemical, physicochemical and practical aspects. Arch. Oral Biol..

[60] Derrien M., van Passel M.W., van de Bovenkamp J.H., Schipper R., de Vos W., Dekker J. (2010). Mucin-bacterial interactions in the human oral cavity and digestive tract. Gut Microbes.

[61] Ito F., Yamada S., Mizuno Y., Sugihara N., Chen L. (1988). Correlation between viscosity and sialic acid content of whole human saliva. Aichi-Gakuin Dent. Sci..

[62] Coles J.M., Chang D.P., Zauscher S. (2010). Molecular mechanisms of aqueous boundary lubrication by mucinous glycoproteins. Curr. Opin. Colloid Interface Sci..

[63] Milton J.C., Hill-Smith I., Jackson S.H. (2008). Prescribing for older people. BMJ.

