# Supplementary figures and images for: Reduced Salivary Mucin Binding and Glycosylation in Older Adults Influences Taste in an In Vitro Cell Model
Source: Nutrients. 2019 Sep 24;11(10):2280. doi: 10.3390/nu11102280 (PMC6835954; doi:10.3390/nu11102280)

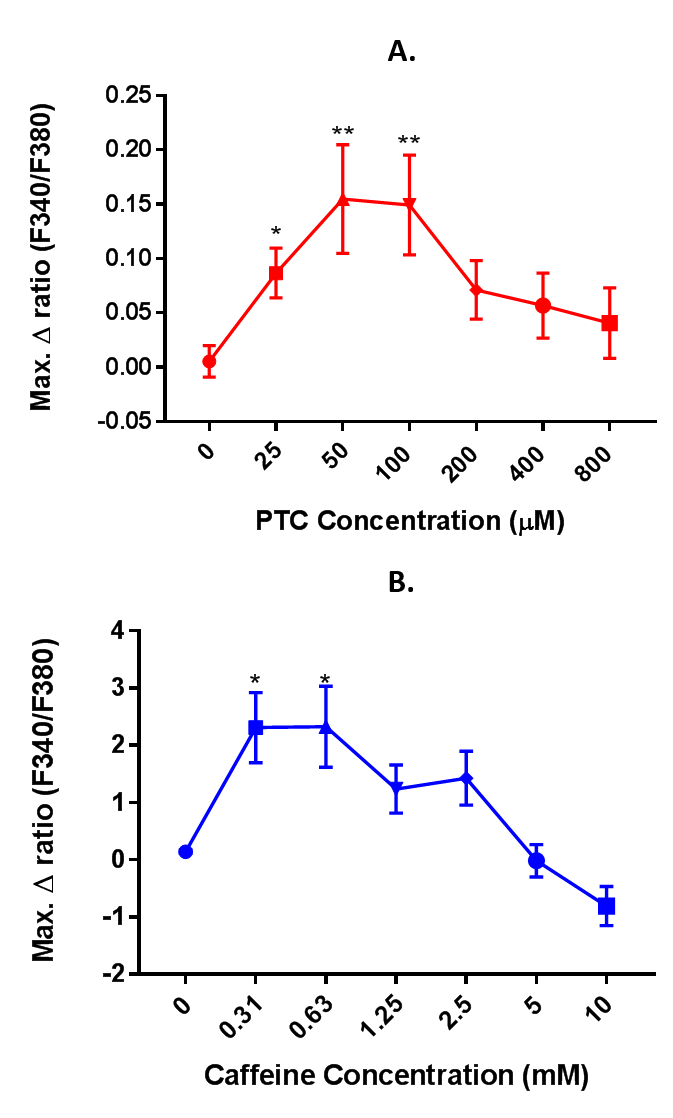

Supplement: Supplementary file 1 [file nutrients-11-02280-s001.zip › Figure S9.tif]

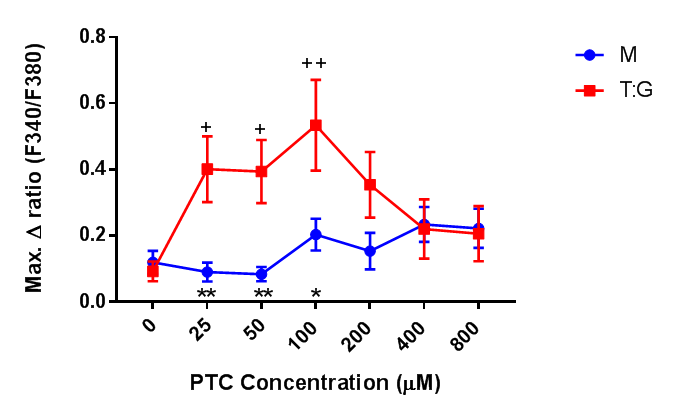

Supplement: Supplementary file 1 [file nutrients-11-02280-s001.zip › Figure S8.tif]

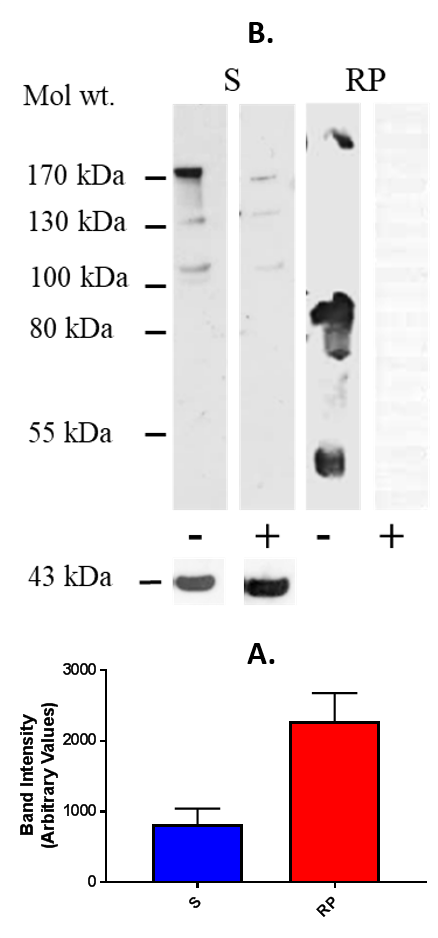

Supplement: Supplementary file 1 [file nutrients-11-02280-s001.zip › Figure S7.tif]

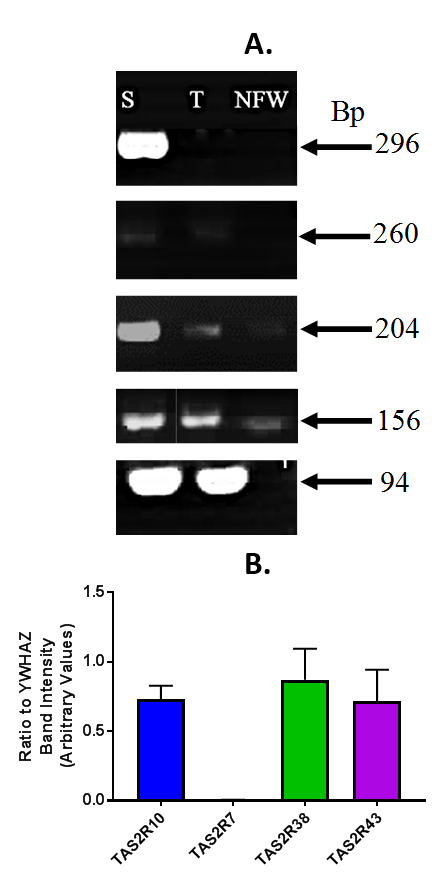

Supplement: Supplementary file 1 [file nutrients-11-02280-s001.zip › Figure S6.tif]

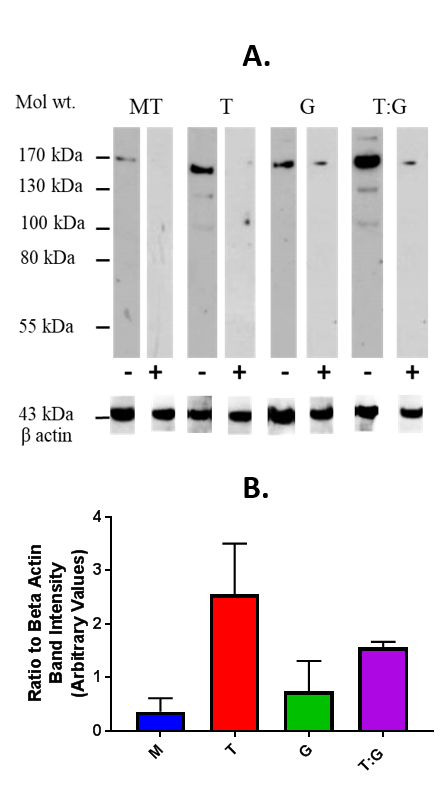

Supplement: Supplementary file 1 [file nutrients-11-02280-s001.zip › Figure S5.tif]

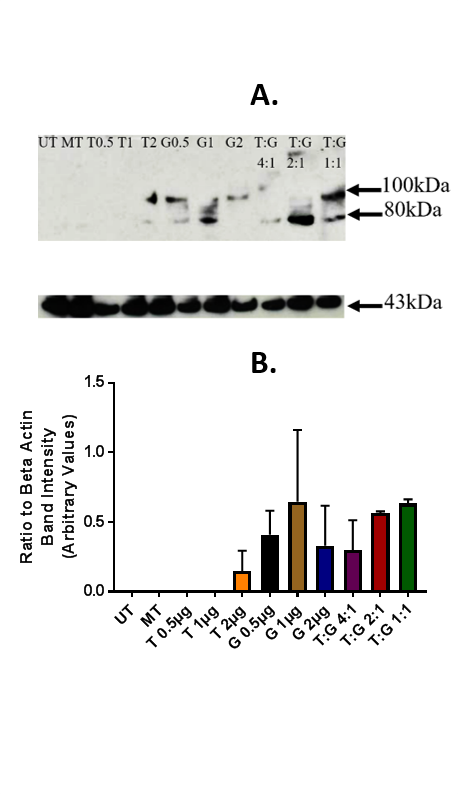

Supplement: Supplementary file 1 [file nutrients-11-02280-s001.zip › Figure S4.tif]

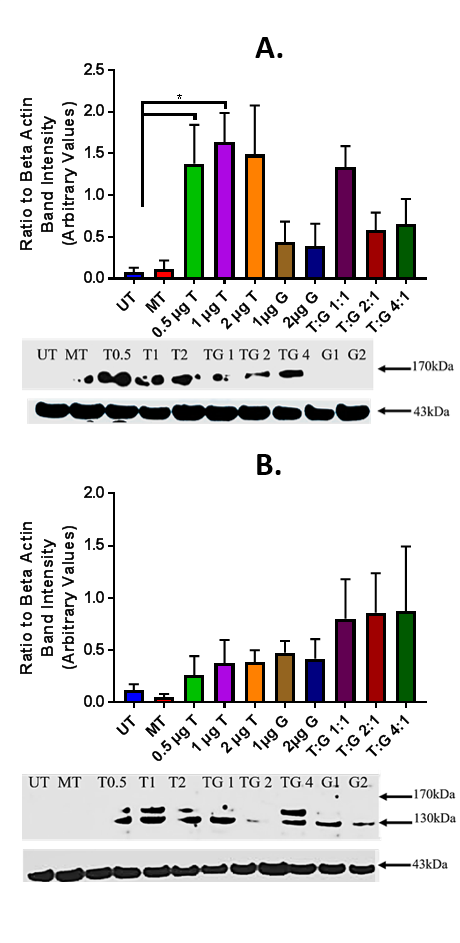

Supplement: Supplementary file 1 [file nutrients-11-02280-s001.zip › Figure S3.tif]

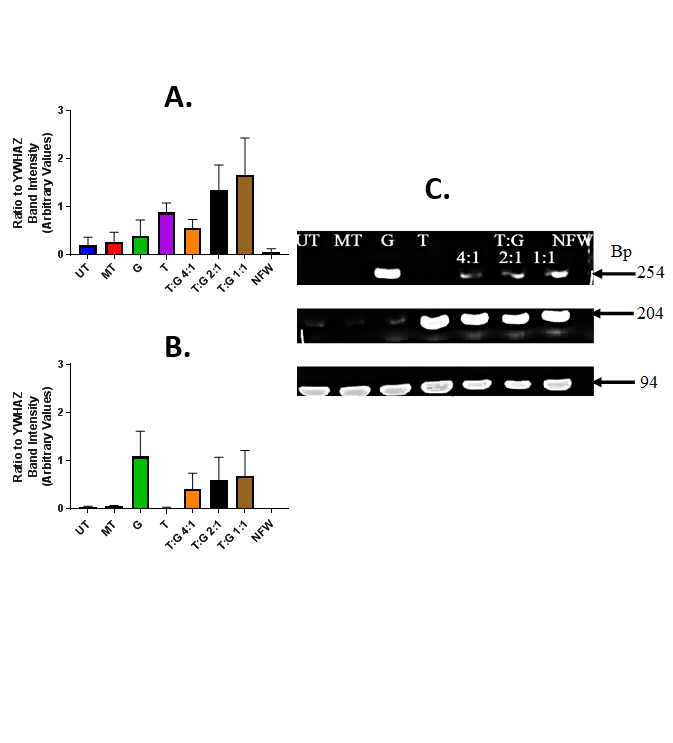

Supplement: Supplementary file 1 [file nutrients-11-02280-s001.zip › Figure S2.tif]

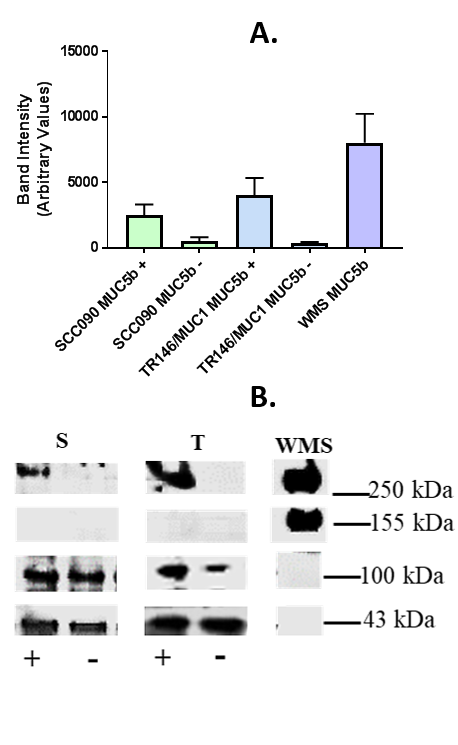

Supplement: Supplementary file 1 [file nutrients-11-02280-s001.zip › Figure S1.tif]
